# Like/Dislike Prediction for Sport Shoes With Electroencephalography: An Application of Neuromarketing

**DOI:** 10.3389/fnhum.2021.793952

**Published:** 2022-01-06

**Authors:** Li Zeng, Mengsi Lin, Keyang Xiao, Jigan Wang, Hui Zhou

**Affiliations:** ^1^School of Business, Hohai University, Nanjing, China; ^2^College of Environment, Hohai University, Nanjing, China; ^3^School of Automation, Nanjing University of Science and Technology, Nanjing, China

**Keywords:** neuromarketing, electroencephalography, machine learning, brain asymmetry, preference prediction

## Abstract

Neuromarketing is an emerging research field for prospective businesses on consumer’s preference. Consumer’s preference prediction based on electroencephalography (EEG) can reliably predict likes or dislikes of a product. However, the current EEG prediction and classification accuracy have yet to reach ideal level. In addition, it is still unclear how different brain region information and different features such as power spectral density, brain asymmetry, differential entropy, and Hjorth parameters affect the prediction accuracy. Our study shows that by taking footwear products as an example, the recognition accuracy of product likes or dislikes reaches 94.22%. Compared with other brain regions, the features of the frontal and occipital brain region obtained a higher prediction accuracy, but the fusion of the features of the whole brain region could improve the prediction accuracy of likes or dislikes even further. Future work would be done to correlate the EEG-based like or dislike prediction results with product sales and self-reports.

## Introduction

Neuromarketing is an emerging interdisciplinary research area that aims to understand biology of consumer’s behavior by integrating neuroscience with marketing, which can decipher consumers’ unrevealed preferences, motivations, and decisions by measuring their physiological and neural signals (Ariely and Berns, 2010; Morin, 2011; Aldayel et al., 2020; Bazzani et al., 2020). It is estimated that neuromarketing has market potential of 400 billion dollars (Khurana et al., 2021). Conventional marketing provides only relative analysis of consumer’s response, which relies on conducting surveys, interviews, running focus groups, and field trials for collecting consumer’s feedback. These analysis approaches suffer limitations due to high cost, time requirement, and untrustworthy information. Besides, conventional approaches have significant inherent weaknesses arising from consumers not always forthcoming about their feelings and preferences. All of these drawbacks would lead to biased or inaccurate conclusions (Khushaba et al., 2012; Boksem and Smidts, 2015). Compared with conventional marketing research techniques, neuromarketing empowers researchers to capture consumers’ intricate neural processes to a range of marketing stimuli with moment-to-moment neural data, allowing to forecast consumer’s decision-making, like–dislike, and purchase decisions with greatly improved accuracy (Venkatraman et al., 2012; Barnett and Cerf, 2017; Bell et al., 2018; Goto et al., 2019).

In neuromarketing, neural signal recording techniques are commonly used to directly measure consumer’s brain responses to the marketing stimuli (Ariely and Berns, 2010; Sánchez-Fernández et al., 2021). Popular noninvasive neuroscientific techniques to analyze and understand consumer’s behavior include brain imaging technologies such as electroencephalography (EEG), magneto-encephalography (MEG), functional magnetic resonance imaging (fMRI), functional near-infrared spectroscopy (fNIRS), and various physiological parameters (e.g., heart rate and respiratory rate) (Khurana et al., 2021; Qing et al., 2021). For neuromarketing, EEG has several benefits that other physiological signals lack, such as high temporal resolution for detecting brain activity changes at low cost, equipment portability. Because it can be easily employed in real-time marketing environments, it is desirable to use EEG to capture electrical brain activity and assess marketing stimuli to build preference prediction system in the neuromarketing research (Aldayel et al., 2020).

For companies and advertisers, it is of great significance to successfully predict consumer’s preference of specific products. They can reduce inventory, increase profits, grow customer loyalty, and satisfaction and improve branding by competently avoiding the production of unpopular or undesirable products. Consumer’s preferences of footwear products were proficiently predicted using EEG data as compared to self-report-based predictions (Baldo et al., 2015). Yılmaz et al. (2014) researched consumers’ likes and dislikes of footwear products using EEG signals and revealed the particularly different channels and frequencies of likes and dislikes. In neuromarketing, power spectral density (PSD) is one of the most common feature extraction method (Khushaba et al., 2012; Yılmaz et al., 2014). Some studies theorize that the PSD of EEG signals can be used to identify the likes and dislikes of products (Golnar-Nik et al., 2019). Additionally, some studies presume that features and parameters of frontal brain asymmetry, such as approach-withdrawal (AW) index, effort index, choice index, valence, can expertly identify product preference of consumers (Cartocci et al., 2017; Ramsøy et al., 2018; Aldayel et al., 2020, 2021). Differential entropy (DE) and Hjorth parameters are commonly used EEG features in emotion recognition (Chen et al., 2019; Joshi and Ghongade, 2022). However, as far as we know, the use of these features in neuromarketing has seldom been reported.

It is often challenging for neuromarketing researchers to choose the appropriate characteristics from these many EEG characteristics to accurately predict product likes or dislikes (Bell et al., 2018). Therefore, it is necessary to carry out the research to compare the classification accuracy of these features, so that further studies in the field of neuromarketing can be done to apply to more product like and dislike cases.

The purpose of this study is to design a product like or dislike prediction system based on EEG using commonly worn sport shoes as product example, so that comparison of the characteristics of consumer’s preference of commonly used EEG in published literature can be made and also to compare the classification accuracy of these features. This can help neuromarketing scholars to design a classification and prediction system based on EEG. The contributions of this research are as follows:

(1) Developing a consumer’s like or dislike prediction system based on EEG to achieve high classification accuracy by taking sport shoes as an example.
(2) Study the influence of different EEG characteristic parameters such as different brain locations, PSD features, brain asymmetry features, Hjorth features, and DE features and compare their classification accuracies.

Based on the abovementioned criteria, our study looks to implement method and provide test results of the EEG-based shoe like or dislike prediction system in the following chapters. To attain our goal, EEG-based preference detection experiments were conducted. During the experiment, electrical brain activity of 15 subjects aged between 22 and 39 was recorded. Subjects were presented with pictures of 25 different sport shoes one by one and were asked to decide whether they liked or not by pressing 1 on keyboard for like and 2 on keyboard for dislike. In session II, the details of participants, trial design, experimental equipment, and machine learning classifier framework are explained. In session III, the PSD, brain asymmetry, Hjorth parameter, and DE were extracted as features. Machine learning classifiers SVM and KNN are used, and finally, the system is evaluated by measuring the accuracies of the classifier.

## Materials and Methods

### Subjects

Fifteen healthy subjects (nine men and six women, 22–39 years old, all right-handed) who were students of Nanjing University of Science and Technology participated in the experiment. All participants reported normal hearing and the absence of any neurological disorders. They were informed about the purpose and experimental procedure of the study. Both genders were participated in the experiment to explore possible influence of gender in results. The recruitment of subjects and the experimental protocols were approved by the Ethics Committee for Human Research, Nanjing Brain Hospital Affiliated to Nanjing Medical University.

### Experiment Protocol

**FIGURE 1:**
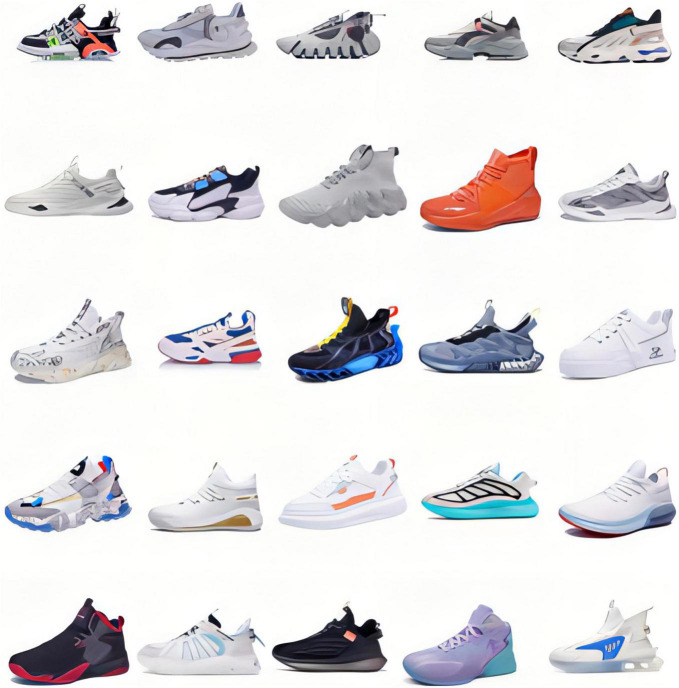
Images of 25 different sport shoes used in the experiment.

A computerized task has been designed to investigate the ability of EEG power to distinguish between consumer’s preferences among subjects. Participants were asked to make a decision for liking or disliking of specific product. According to 2020 online shopping report of China, the most favorable commodities among online consumers are daily necessities, clothing and shoes. Therefore, for this study, pictures of 25 different sport shoes under the Chinese Li-Ning brand were selected in the experiment as shown in Figure 1.

**FIGURE 2:**
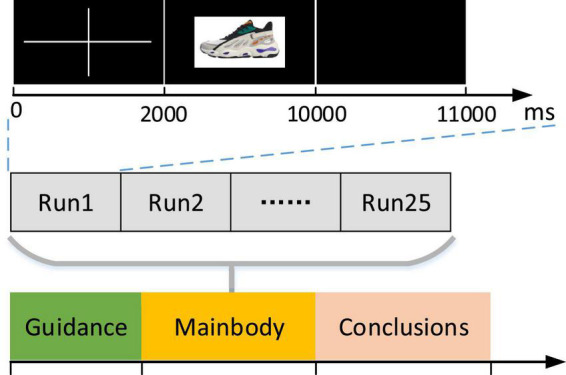
The experimental protocol. The duration of each run was 11 s, a fixation cross was shown for 2 s, and then, a picture of specific shoe appeared for 8 s, followed by a rest of 1 s.

The experimental paradigm is implemented using E-Prime software. Figure 2 displays the entire process of experimental protocol. The main part of the experiment is divided into 25 runs (iterations/epochs). Each run lasts for 11 s, and it includes prompting to focus attention for 2 s, observing image of specific shoe for 8 s, and resting for 1 s. Each round will randomly present a shoe image without repetition, and the subjects were asked to look at the product image. In the displayed 8 s, subjects make a decision on whether they like the shoe or not and record their decision through the keyboard by pressing “1” for like and “2” for dislike.

### Data Acquisition and Data Preprocessing

During the experiment, a 32-channel EEG acquisition device, BrainAmp Amplifier (Brain Product, Gilching, Germany) and active Ag/AgCl electrodes (actiCAP, Brain Product, Germany), was used to collect the EEG signals of the subjects. For each run (epoch), the EEG data are recorded from 22 silver chloride electrodes placed on subject’s head in the international 10–20 system. The specific placement locations are as follows: FP1, FPz, FP2, F7, F3, Fz, F4, F8, T7, C3, Cz, C4, T8, TP9, TP10, P7, P3, Pz, P4, P8, O1, O2, with FPz as the ground electrode and Fz as the reference electrode. The conductive paste is used to make the contact impedance between the electrode and the scalp less than 5 kΩ, the sampling rate is set to 500 Hz, and the bandpass filter was set at 0.03–70 Hz.

To accurately collect effective EEG data, the participants were asked to ensure adequate sleep before the experiment, stimulants such as cigarettes, alcohol, coffee, and strenuous exercise should be avoided prior to testing, and hair should be cleaned. The environment was kept quiet during the experiment. The participants sat in a comfortable chair, looked at the screen squarely, kept their mind and body in relaxed state, maintained their posture, and reduced the number of eye blinks.

Brain analyzer is used to preprocess raw EEG data recorded in the experiment, including re-referencing, filtering, removing artifacts, segmenting, and reducing the sampling rate. First, we re-referenced the data. The current electrode caps usually use Fz as the reference electrode, but in the analysis of EEG data, the reference electrode needs to be replaced according to the experimental requirements. The reference electrodes used in this analysis are bilateral mastoid TP9 and TP10. Then, we filtered the data. The raw EEG signals were bandpass filtered using a 4th-order Butterworth filter set at 0.5–40 Hz to filter out high-frequency noise. We need to carry out the necessary artifact removal operation for EEG signal. As the artifact signals caused by the device or the subject’s actions will cause errors in subsequent data processing and experimental results, this article uses independent component analysis (ICA) to correct physiological artifacts such as electrooculogram (EOG). Then, we need to segment the data into likes and dislikes. According to the markers formed on the EEG data of the subjects’ choice reaction to the product during the experiment, the data of the state of like and dislike are extracted separately. Finally, the data are sampled down. To reduce the amount of data and increase the calculation speed, the sampling rate is reduced to 256 Hz.

### Feature Extraction

To select appropriate features, different feature extraction technologies are used and compared for predicting like and dislike of a shoe product. The EEG features of PSD, brain asymmetry, DE, and Hjorth parameter were chosen in this study. In neuromarketing, PSD is one of the most common feature extraction methods (Khushaba et al., 2012; Yılmaz et al., 2014; Golnar-Nik et al., 2019). The brain asymmetry-based preference indices such as approach-withdrawal (AW) index, valence, choice index, and effort index are also used as features to predict consumer’s preference (Aldayel et al., 2020, 2021). Besides, EEG features such as DE and Hjorth parameters have generally been used in some EEG-based applications (Chen et al., 2019; Joshi and Ghongade, 2022). However, as far as we know, the use of these features in neuromarketing has seldom been reported.

#### Power Spectral Density

The PSD is an indicator of power in a certain signal in terms of frequency. The Welch method is used to estimate the PSD of the EEG signal, and the PSD of the time series is calculated as below (Welch, 1967):


(1) $${\hat{S}}_{XX}\left(k\right)=\frac{1}{N}\sum_{n=1}^{N}|X_{n}(k){|}^{2}$$

where *X*_*n*_(*k*) is the Fourier transform of the time series × corresponding to the *n*th segment and the *k*th frequency point after windowing. In this study, the relative power in the four frequency bands of δ (0.5–4 Hz), θ (4–8 Hz), α (8–13 Hz), and β (13–30 Hz) is calculated based on the PSD of each channel data.

#### Brain Asymmetry

The AW index of frontal alpha asymmetry estimates desire and motivation as alpha’s higher activation in the left frontal cortex. We can measure the AW scores using electrodes F4 and F3 to find the difference between the right and left PSD divided by their amounts according to Eq. 2 (Touchette and Lee, 2017).


(2) $$\mathrm{AW}\mathrm{index}=\frac{\text{α}\left(F4\right)-\text{α}\left(F3\right)}{\text{α}\left(F4\right)+\text{α}\left(F3\right)}$$

The effort index measures effort and cognitive processing as higher theta activation in the prefrontal cortex. We used the following equation to calculate the effort index (Aldayel et al., 2021).


(3) $$\mathrm{Effort}\mathrm{index}=\frac{\text{θ}\left(F4\right)-\text{θ}\left(F3\right)}{\text{θ}\left(F4\right)+\text{θ}\left(F3\right)}$$

The choice index is defined in Eq. 4. The choice index can be calculated for each band individually using electrodes pairs of left and right counterparts for each lobe according to Eq. 4 (Moon, 2013; Ramsøy et al., 2018).


(4) $$\mathrm{Choice}\mathrm{index}=\frac{\text{log}\left({\text{Electrode}}_{left}\right)-\text{log}\left({\text{Electrode}}_{right}\right)}{\text{log}\left({\text{Electrode}}_{left}\right)+\log\left({\mathrm{Electrode}}_{right}\right)}$$

The valence measures positive emotion as left frontal activation in alpha and beta bands. In this study, we computed the values of valence using Eq. 5 (Al-Nafjan et al., 2017).


(5) $$\text{Valence}=\frac{\text{α}\left(F4\right)}{\text{β}\left(F4\right)}-\frac{\text{α}\left(F3\right)}{\text{β}\left(F3\right)}$$

#### Differential Entropy

Differential entropy is defined in Eq. 6, where *p*(*x*) represents the probability density function of continuous information (Shi et al., 2013).


(6) $$DE=-\int_{a}^{b}p\left(x\right)\text{log}\left(p\left(x\right)\right)\text{d}x$$

For EEG signal with a specific length that approximately follows a Gaussian distribution, its DE is expressed as follows:


(7) $$\begin{array}{c} DE=-\int_{a}^{b}\frac{1}{\sqrt{2\pi \sigma_{i}^{2}}}{\text{e}}^{-\frac{{\left(x-\text{μ}\right)}^{2}}{2{\text{σ}}_{i}^{2}}}\log\left(\frac{1}{\sqrt{2\pi \sigma_{i}^{2}}}e^{-\frac{{\left(x-\text{μ}\right)}^{2}}{2{\text{σ}}_{i}^{2}}}\right)\text{d}x\\ =\frac{1}{2}\log\left(2\pi e\sigma_{i}^{2}\right) \end{array}$$

#### Hjorth Parameters

Hjorth introduced the Hjorth parameters to describe the EEG signal in the time domain, including the following three characteristics, which are activity, mobility, and complexity (Hjorth, 1970):

Activity measures the degree of deviation of the signal amplitude:


(8) $$Activity=\frac{1}{N}\sum_{n=1}^{N}{\left(s(n)-\mu_{s}\right)}^{2}$$

Mobility measures the changes in slope:


(9) $$Mobility=\sqrt{\frac{var\left(s^{{}^{\prime }}\left(n\right)\right)}{var\left(s\left(n\right)\right)}}$$

Complexity measures how many standard slopes are there on an amplitude:


(10) $$Complexity=\frac{Mobility\left(s^{{}^{\prime }}\left(n\right)\right)}{Mobility\left(s\left(n\right)\right)}$$

where μ_*s*_ represents the average value of the signal, *s*′(*n*) represents the first derivative of the signal, and *var*(⋅) represents the variance.

### Classification and Statistical Analysis

**FIGURE 3:**
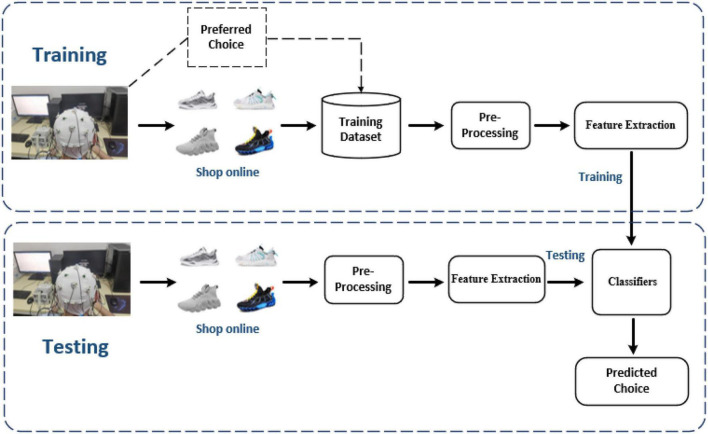
Flow chart of the proposed electroencephalography (EEG)-based consumer’s preference prediction model.

This study implements k-nearest neighbor (KNN) and support vector machine (SVM) classifiers to distinguish the EEG characteristics of consumer’s preference between likes and dislikes based on KNN with Euclidean distance [KNN(E)], KNN with cosine distance [KNN(C)], SVM with radial basis kernel [SVM(R)], and SVM with polynomial kernel [SVM(P)] since these are commonly used machine learning methods. During the experiment, the EEG signals are collected when the subjects watch pictures different sport shoes. The 10-fold crossvalidation was performed, and all subjects’ data were collected and randomly split into training set (90%) and test set (10%). At the same time, the data of two data sets are preprocessed and extracted. Among the features extracted were PSD, brain asymmetry, DE, and Hjorth parameters. Then, the parameters of the classifier model are trained by the feature data and labels in the training set, and then, the performance of the trained model is evaluated by the feature data and labels in the test set, as shown in Figure 3.

The classification accuracy was defined as:


(11) $$Accuracy=\left(\frac{N_{correct}}{N_{total}}\right)\times 100\%$$

where *N*_*total*_ and *N*_*correct*_ are defined as the total number of samples to be classified and the number of correct samples. The final classification accuracy was the average of 10 repetitions.

For statistical analysis of difference in power of EEG between like and dislike decision, two-sample *t*-tests are performed by calculating the power of different frequency bands. The *p*-value (≤0.05) from two-sample *t*-tests represent the significant contrast between liked and disliked decisions.

## Results

### Most Liked or Disliked Sport Shoes

**FIGURE 4:**
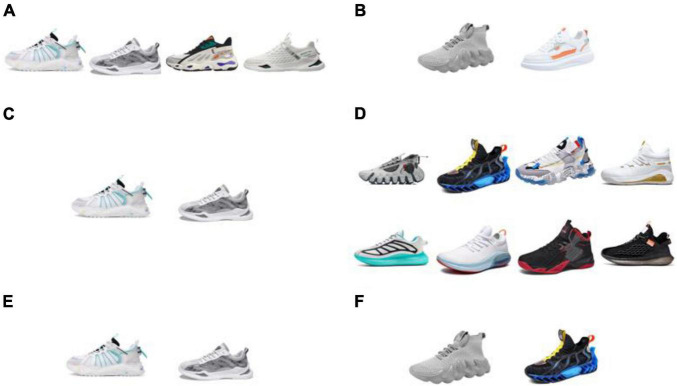
Most liked and disliked shoes among different subject types (men, women, and all). Panel **(A)** are the most liked shoes among male subjects, panel **(B)** are the most disliked shoes among male subjects, panel **(C)** shows most liked shoes among female subjects, panel **(D)** shows most disliked shoes among female subjects, panel **(E)** are overall most liked shoes among all subjects, and panel **(F)** are overall most disliked shoes among all subjects.

Figure 4 displays the most liked and disliked shoes among the following subject groups: male subjects only, female subjects only, and all subjects. Figure 4A displays most liked shoes among male subjects. The shoe farthest on the left is liked by seven male subjects, and the other three are equally liked by six male subjects. Almost all male subjects disliked the shoe styles in Figure 4B. The two shoe styles in Figure 4C were equally liked by five female subjects, respectively, and all of the female subjects disliked the 8 shoe styles in Figure 4D. Finally, we obtained the two shoes which all subjects (men and women combined) like and dislike the most. In Figure 4E, the shoes that were liked by 12 and 11 subjects are shown from left to right. In Figure 4F, the two most disliked shoes are shown, each of which was liked by only one subject.

### Power Spectral Density Analysis

**FIGURE 5:**
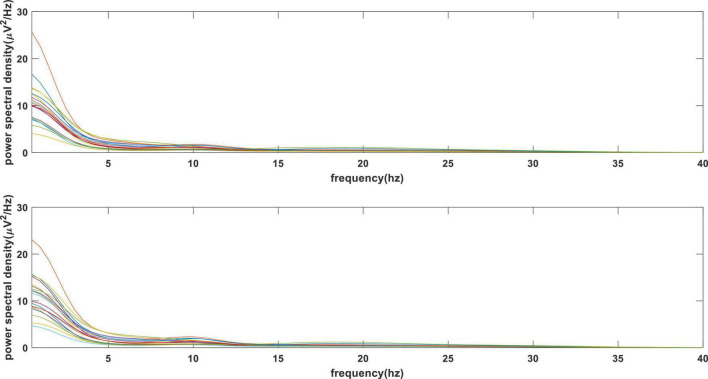
Average power spectral density (PSD) vs. frequency values of all channels. *Upper panel* displays liked case and *lower panel* shows disliked cases.

The PSD of 0.5–40 Hz was calculated for 2,250 samples (25 runs × 15 subjects × 6 segments of 8-s data) of all subjects. The results were averaged according to the label. Figure 5 shows the PSD result where the upper and lower parts represent the like and dislike.

**FIGURE 6:**
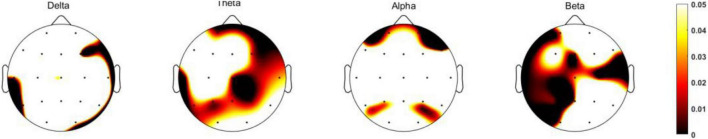
The brain topography of the *p*-value distribution for EEG power difference between like and dislike decision in different frequency bands.

Figure 6 represents the significant power difference between like and dislike choices made by subjects. In the delta frequency band, significant EEG power difference between like and dislike can be observed in the right frontal, left temporal, right temporal, and right occipital regions. For the theta frequency band, the significant power difference is concentrated in temporal, parietal, central, and frontal in asymmetry manner. Significant power difference in the alpha frequency band is concentrated in the frontal (symmetry) and parietal areas. In addition, the significant power difference in the beta frequency band is found in the frontal, temporal, central, parietal, and occipital regions. These findings are similar to other studies (Golnar-Nik et al., 2019).

### Classification Result

**FIGURE 7:**
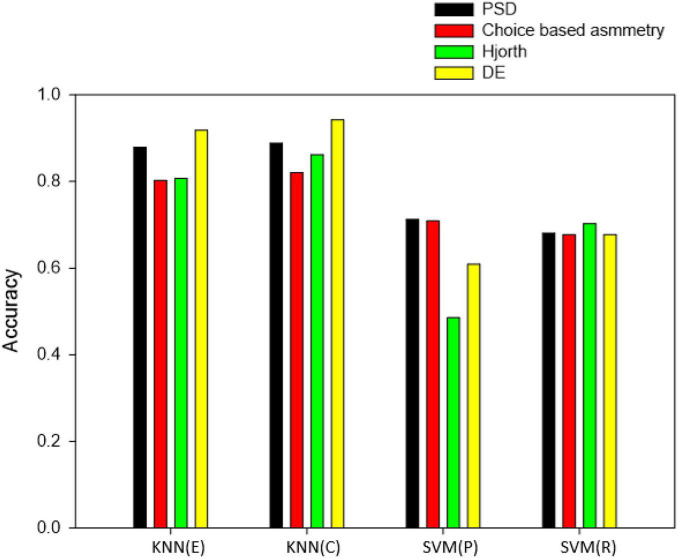
Classification accuracy of four classifiers based on different feature sets.

Figure 7 shows the classification results of four different classifiers based on four different feature sets. It can be observed from the classification accuracy that the two classifiers KNN(C) and KNN(E) give better performance than the two classifiers SVM(P) and SVM(R) based on PSD features, choice-based symmetry features, Hjorth parameter features, and the DE features. KNN(C) classifier gives the best results where the accuracy is 88.85% for PSD features, 82.04% for choice-based asymmetry features, 86.17% for Hjorth features, and 94.22% for DE features. Note that 16 channels were used for choice-based asymmetry features.

**FIGURE 8:**
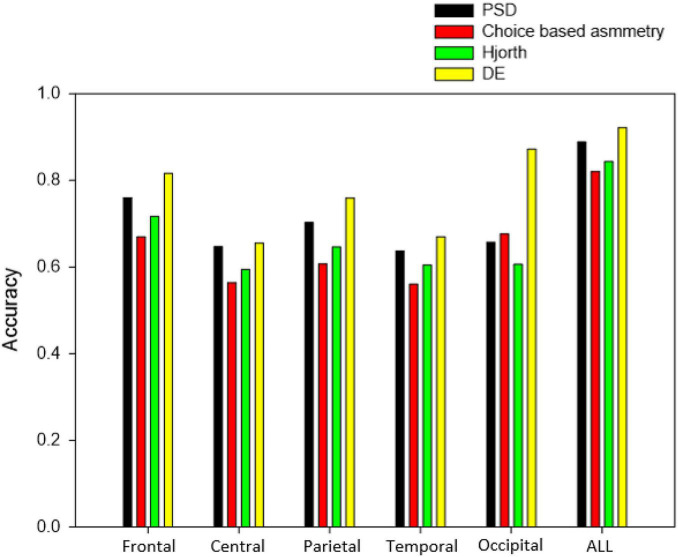
Classification accuracy of different brain lobes based on different feature sets.

Figure 8 shows the classification results of different brain lobes based on different feature sets, and the experimental results shown below are based on the KNN(C) classifier. The results showed that the difference in like and dislike of shoe products was most apparent in the DE features of the occipital locations (87.16%). However, fusion of all brain region areas increases classification accuracy of all four feature sets.

**FIGURE 9:**
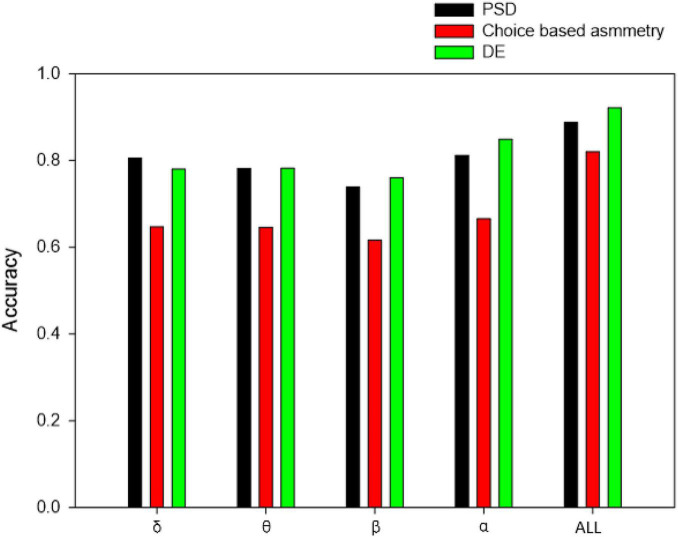
Classification accuracy of different frequency bands based on different feature sets.

Figure 9 shows the classification results of different frequency bands based on different feature sets, and the experimental results shown below are based on the KNN(C) classifier. Compared with other frequency bands, the results show that alpha bands of signals are slightly more conducive to the distinction between consumer’s likes and dislikes. Besides, the features of other frequency bands can also be used to achieve like and dislike classification. The fusion of all frequency bands resulted in improved classification accuracies of like and dislike prediction.

**FIGURE 10:**
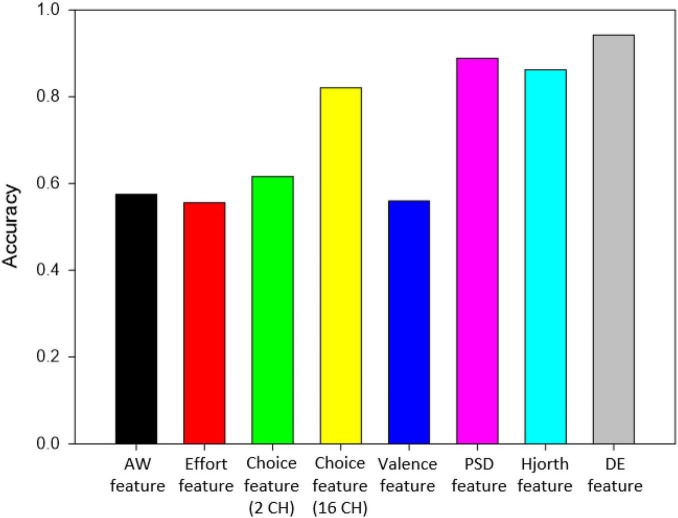
Classification accuracy of different EEG features.

Figure 10 shows comparison of the classification accuracies of features of different EEG indices based on the KNN(C) classifier. It is observable that all four EEG asymmetry-based features (AW index, effort index, choice index, and valence) provide similar classification accuracy when the same channels were used (F4 and F3). However, 16 channel choice index-based features give higher accuracy than 2 channel choice features and rest of the EEG index-based features. Nevertheless, features of PSD (88.85%), Hjorth (86.17%), and DE (94.22%) provide highest classification accuracy and noticeably greater than the rest.

## Discussion

In this research, an EEG-based consumer’s preference prediction system is proposed to predict whether consumers’ like or dislike particular product. EEG data were recorded from 15 recruited subjects whereas they watched pictures of 25 different designs of sport shoes and made decisions about their likes and dislikes of shoes that were shown. The EEG-based consumer’s like or dislike prediction system demonstrated in this work achieved a classification accuracy of 94.22% using DE features.

Our research weighs up the effects of the characteristics of different brain regions on classification accuracy. It can be observed from the *p*-value distribution of EEG power that the frontal and occipital regions play important part in consumer’s like and dislike decision-making process. Furthermore, the features extracted from those two regions also may have higher classification accuracy than the other brain regions.

Electroencephalography-based preference indices such as AW index, effort index, choice index, and valence are often used to measure the response of subjects to market stimuli. However, the classification accuracy obtained from the features extracted from these indices is seldom reported. Our research finds that classification accuracy of these indices is similar to accuracy of choice index being slightly higher. Increasing the number of channels sees increase in classification accuracy among all EEG features, as evidenced by the difference between 2 channel and 16 channel choice index features. Our research implemented a method that demonstrated extracted DE features which were able to obtain greatly higher classification accuracy (>90%) for both KNN(C) and KNN(E) classifiers. Besides, combining features of all frequency bands see improvements in classification accuracy.

However, there are some limitations in this study. First, only product images were used as marketing stimuli, and other factors such as brand, ratings, and price were not considered in the experiment. Second, there were only 15 subjects of college students recruited in the experiment for consumer’s preference prediction of sport shoes. Increasing the number of subjects of different ages, incomes, and social status would help to investigate the influence of different factors (brand and ratings, etc.) on consumer’s preferences (Golnar-Nik et al., 2019). Furthermore, the like or dislike prediction accuracy was not correlated with sales of product or self-reports in the study. By comparing with conventional marketing research methods such as sales or self-reports, the results of preference prediction based on EEG could be made more reliable. More subjects would be recruited in the future to investigate the influence of some factors, such as brand and price, on the prediction results of consumer’s preferences, while conducting correlation analysis with product sales and self-reports, etc.

## Conclusion

This manuscript proposes a consumer’s preference prediction system based on EEG by taking sport shoes as an example. The results show that the classification accuracy of 94.22% is achieved based on the DE features. The method proposed in this study can be used for product preference prediction. Furthermore, the number of EEG channels and recording location can be optimized to make the system easy-to-use and time-effective. In the future, the product like or dislike prediction results would be correlated with product sales and self-reports to make the results of EEG-based preference prediction more reliable.

## Data Availability Statement

The raw data supporting the conclusions of this article will be made available by the authors, without undue reservation.

## Ethics Statement

The studies involving human participants were reviewed and approved by Nanjing Brain Hospital Affiliated to Nanjing Medical University. The patients/participants provided their written informed consent to participate in this study.

## Author Contributions

LZ, HZ, and JW initiated and supervised the research project. LZ, ML, and KX carried out the research, analyzed the results and prepared the figures. LZ, ML, and HZ wrote part of the manuscript. All authors contributed to the article and approved the submitted version.

## Conflict of Interest

The authors declare that the research was conducted in the absence of any commercial or financial relationships that could be construed as a potential conflict of interest.

## Publisher’s Note

All claims expressed in this article are solely those of the authors and do not necessarily represent those of their affiliated organizations, or those of the publisher, the editors and the reviewers. Any product that may be evaluated in this article, or claim that may be made by its manufacturer, is not guaranteed or endorsed by the publisher.
